# The beneficial effect of Alpha-lipoic acid supplementation as a potential adjunct treatment in episodic migraines

**DOI:** 10.1038/s41598-021-04397-z

**Published:** 2022-01-07

**Authors:** Mahnaz Rezaei Kelishadi, Amirmansour Alavi Naeini, Fariborz Khorvash, Gholamreza Askari, Zahra Heidari

**Affiliations:** 1 Department of Community Nutrition, School of Nutrition and Food Science, Isfahan University of Medical Sciences, Isfahan, Iran; 2 Department of Neurology, School of Medicine, Isfahan University of Medical Sciences, Isfahan, Iran; 3 Department of Biostatistics and Epidemiology, School of Health, Isfahan University of Medical Sciences, Isfahan, Iran

**Keywords:** Diseases, Neurology

## Abstract

The current study was performed to evaluate the effects of alpha-lipoic acid (ALA) supplementation on lactate, nitric oxide (NO), vascular cell adhesion molecule-1 (VCAM-1) levels, and clinical symptoms in women with episodic migraines. Considering the inclusion and exclusion criteria**,** ninety-two women with episodic migraines participated in this randomized, double-blind, placebo-controlled, parallel-design trial. The participants were randomly assigned to receive either 300 mg/day ALA or placebo, twice per day for 12 weeks. The primary outcomes included headache severity, headache frequency per month, and duration of attacks and the secondary outcomes included lactate (a marker of mitochondrial function), NO, and VCAM-1 serum levels were measured at baseline and the end of the intervention. At the end of the study, there was a significant decrease in lactate serum levels (− 6.45 ± 0.82 mg/dl vs − 2.27 ± 1.17 mg/dl; *P* = 0.039) and VCAM-1 (− 2.02 ± 0.30 ng/ml vs − 1.21 ± 0.36 ng/ml; *P* = 0.025) in the ALA as compared to the placebo group. In addition, the severity (P < 0.001), frequency (P = 0.001), headache impact test (HIT-6) (P < 0.001), headache dairy results (HDR) (P = 0.003), and migraine headache index score (MHIS) (P < 0.001) had significantly decreased in the intervention as compared to the control group. No significant changes were observed for NO levels and duration of migraine pains. ALA supplementation can be considered a potential adjunct treatment in patients with migraine due to its improving mitochondrial and endothelial functions and clinical symptoms.

## Introduction

Migraine is defined as a common, chronic, throbbing, weakening neurological disease, accompanied by a serious one-sided headache, nausea, vomiting, and photophobia^1,2^. The prevalence of migraine worldwide and in the United States is estimated to be 14.7%^3^ and 12%^4^, respectively. Its incidence is also reported to be about 14% among Iranian adults^5^. It should be noted that migraine is more common in women than in men (about three-fold). It also occurs with higher intensity in women^6^. Migraine headaches are divided into episodic (with less than 15 days/month of headaches) and chronic (with more than or equal to 15 days/month of headache for > 3 months, with migraine symptoms on ≥ 8 days/month)^7^. According to the International Headache Society (IHS) criteria, two main classes of migraine are defined: migraine with aura (MA) and migraine without aura (MwoA)^7^. Patients with MA have a combination of optical, sensual, language, or motor function symptoms that are completely reversible^8^. The exact pathophysiological mechanisms of migraine are not completely understood. According to studies, several hypotheses have been proposed in the pathogenesis of migraine^9,10^. One of the recognized hypotheses on migraine is hypoxia or mitochondrial dysfunction^11^; in addition, migraines can be caused by factors such as nitric oxide hypersensitivity and abnormal cortical activity^11^.

Phosphorus magnetic resonance spectroscopy (31P-MRS) studies have displayed variations in mitochondrial energy metabolism in the brains of people with migraine^12,13^. Also, a significant reduction of adenosine triphosphate (ATP) has been reported in the medial occipital lobe of the brain in MwoA patients^11^. Mitochondrial dysfunction and decreased oxygen metabolism can be described as the cause of vascular and neuronal dysfunction in migraine^14,15^. Hypoxia and mitochondrial dysfunction increase lactate levels in the brain of healthy^16,17^, MA and MwoA subjects^18,19^. In some previous studies, elevated serum lactate levels have been reported in patients with migraine (both MA and MwoA) compared with control groups^18,20,21^. In fact, elevated lactate reflects mitochondrial dysfunction, which may play an important role in migraine^19,20^. When hypoxia affects tissue, endothelial cells release nitric oxide (NO), consequently increasing the vasodilatation response and oxygen supply to tissues^22^. Endothelial dysfunction has been exposed in MA and MwoA subjects^22–29^. Past studies have reported increased cerebral blood flow (CBF) in migraine patients^30^. Following elevated CBF, NO production in endothelial cells increases as a response to shear forces and leads to flow-mediated dilation (FMD); thus, it can increase inflammatory responses^28,31,32^. Increased NO levels have been demonstrated in both migraines with and without aura^33^. It has also been shown that NO supersensitivity may be a possible molecular mechanism of migraine pain^34^. NO is involved in stimulating cortical spreading depression (CSD) activity and consequently increasing cellular adhesion molecules (CAMs)^35^. According to previous studies, migraine is associated with an increased risk of several vascular disorders such as ischemic stroke and coronary artery disease^36–38^. The CAMs present on the surface of vascular endothelial cells, vascular smooth muscle cells, and leukocytes, mediate the early stages of atherosclerosis, and are imperative factors in the appraisal of endothelial function^27,39,40^. CAMs enter the systemic blood flow in soluble form. The soluble vascular cell adhesion molecule-1 (sVCAM-1) and soluble intracellular adhesion molecules (sICAM-1) are members of the transmembrane immunoglobulin family and are expressed by cytokine-activated endothelial cells^41^. sVCAMs are involved in inflammation by mediating leukocyte adhesion to vascular cells^41^. CAM serum levels show elevated levels of the intracellular adhesion molecules (ICAM) and vascular cell adhesion molecules (VCAM) in children and young adults with migraine^23,42,43^.

The favorable effects of some metabolism-boosting nutrients such as magnesium, coenzyme Q10, riboflavin, and l-Carnitine in migraine prophylaxis have been proven; these nutrients exert their effects by improving mitochondrial function^44–48^. Alpha-lipoic acid (ALA) is an amphipathic antioxidant, which functions as a co-factor for complex multi-enzymes, such as pyruvate dehydrogenase and ketoglutarate dehydrogenase^49,50^. Several studies have shown that ALA has antioxidant and anti-inflammatory effects^51,52^. ALA exerts its antioxidant power in several ways, which include reducing and regenerating oxidized endogenous antioxidants such as glutathione, vitamin C, and vitamin E, purifying reactive oxygen species (ROS) and nitrogen species (RNS), and modulating the signaling pathways for the nuclear factor kappa B (NF-*κ*B)^53,54^. In addition, the protective effect of ALA on endothelial function has been shown in previous studies^55–57^. Evidence confirms the safety of ALA supplementation in various health conditions in children and adults^58^. One study found that about 90% of migraine patients had abnormally low values of ALA^59^. Few previous studies have surveyed the effect of ALA on the clinical symptoms of migraine^60–62^. To the best of our knowledge, no study has ever evaluated the effect of alpha-lipoic acid supplementation on endothelial factors in patients with migraine. Thus, the existing study was designed to assess the effects of alpha-lipoic acid supplementation on lactate serum levels, nitric oxide, VCAM-1, and clinical symptoms in women with episodic migraines.

## Materials and methods

### Study design

The current study was designed as a randomized, double-blind, placebo-controlled, parallel trial with a 3-month follow-up. This study was conducted based on the ethical guidelines of the 1975 Helsinki Declaration^63^ and was approved by the ethics committee of Isfahan University of Medical Sciences, Isfahan, Iran (IR.MUI.RESEARCH.REC.1399.436), and also registered in the Iranian Registry of Clinical Trials (http://www.irct.ir) as IRCT20161203031212N3. The date of the first clinical trial registration was 9/11/2020.

### Study population

This study included 92 patients with migraine recruited from outpatients referred to Imam Mousa Sadr Clinic, belonging to Isfahan University of Medical Sciences, Isfahan, Iran, from November 2020 to March 2021. Episodic migraine (< 15 headache days/month) was detected by a neurologist on the first visit, in accordance with the criteria of the International Headache Society (IHS)^64^. All patients were female and aged between 20 and 50 years. Patients were selected based on inclusion and exclusion criteria and then randomly allocated to the intervention or control group. Before the study commenced, all subjects had a history of migraine signs for more than 6 months, with at least two attacks per month. At the beginning of the study, informed consent was obtained from the participants. Random assignment was carried out using Permuted Block Randomization (PBR) after age matching (with a 5-year interval). Randomization was performed by blocks of two, and random distributions were conducted by a technician cooperating with the study. During the study, except for the randomization technician, others, including the researchers, patients, and laboratory technicians, were blinded to the random allocations.

### Inclusion criteria

Migraine patients (without aura), with at least 2 attacks per month; headaches lasting between 4 and 72 h; non-menopausal women between 20 and 50 years old; non-alcoholic, non-smoker, non-pregnant, and non-lactating patients; and patients who had been receiving fixed medication for at least 4 weeks before entering the study, all of whom agreed to participate in it.

### Exclusion criteria

Patients with chronic migraine; patients experiencing menopause, pregnancy or lactation; history/presence of any chronic diseases (including cardiovascular disease, diabetes, thyroid disorders, liver diseases, kidney failure, hypertension, and other chronic disorders); malignancies, neurological disorders, and any changes in received medication (in terms of type or dosage); intake of less than 90% supplement and following a diet or workout program during the previous six months; taking antioxidant supplements in the previous 4 weeks and during the study; and intolerance of, or allergy to, ALA.

### Sample size

The sample size was calculated according to the sample size formula recommended for similar trials, which considers the severity of migraine as one of the main consequences of the disease, and based on the previous study (σ equals 1.24) and considering α = 0.05, test power, and effect size were determined to be 80% and 0.2 respectively^48^. A total of 45 individuals per group was required.

### Intervention

After assessing the subjects’ eligibility based on the inclusion criteria and a meeting with a neurologist, a total of 92 patients were included in the present study; they were randomly allocated to the ALA (n = 47) or the placebo group (n = 45). The intervention group received 300 mg/day ALA supplement (Raha Company, Iran) twice a day, for 3 months, and subjects in the control group received the placebo capsules in the same package, dosage, color, and form to ensure a blinded design. The ALA or placebo capsules were coded as A and B in a double-blind method. All the patients and investigators were blinded to the treatment codes. Participants were asked to take capsules 15 min before lunch and before dinner with one glass of water. The subjects were given a list of foods containing tyramine, and they were asked not to consume any such foods during the study. The patients were asked to return to the clinic 40 days after starting the study to receive the second supplement package. Adherence was assessed based on supplement box delivery, counting the remaining capsules, and patient self-report. Adherence to continuous supplementation was also monitored through phone calls with the patients once a week.

### Assessment of anthropometric characteristics, physical activity, and food intake

The anthropometric assessments, including body weight and height, were carried out as dictated by WHO standard processes with the least amount of clothing, and body mass index (BMI) was calculated as weight (kg) divided by the square of height (m^2^). Three-day food records were documented at the beginning and end of the study, and physical activity was recorded at the beginning of the study. Patients were also recommended to maintain their usual physical activity and dietary pattern during the study. Afterward, the described portion sizes in the records were converted to grams using household measures. Dietary intake was analyzed by Nutritionist IV software (First Databank, San Bruno, CA, USA) adapted for Iranian foods. A short form of the international physical activity questionnaire (IPAQ) was used to compute the level of physical activity^65^.

### Primary outcomes: clinical status assessment

A neurologist specified the symptoms of the migraine attacks, for instance, headache severity, frequency per month, and duration. The visual analog scale (VAS) on a 0–10 numeric scale was executed to assess migraine severity^66^. The duration was expressed based on the mean duration (hours) of the migraine attacks. Additionally, the migraine headache index score (MHIS) was considered as the result of headache duration (day) × headache frequency × headache severity.

The headache impact test (HIT-6) is a 6-item questionnaire, an instrument to assess the unfavorable effects of headaches on the daily performance and wellbeing of the patient. The HIT-6 score for each patient ranges from 36 to 78, and lower scores show weaker migraine effects on the patient’s clinical status. A score above 60 points indicates severe impact, 56–59 indicates substantial impact, 50–55 indicates intermediate impact, and 36–49 indicates slight or no impact of migraine on the patient's life^67^. The validity and reliability of the questionnaires had formerly been established for Iranians^68^.

### Secondary outcomes: biochemical measurements

Venous blood samples were collected at the baseline and after the 3 months of intervention, after an overnight fast of 12 h. All the patients were requested to be present in the laboratory on a headache-free day. To separate the serum, whole blood samples were centrifuged at 3000 rpm for 10 min and were stored at − 80 °C until analysis. The serum levels of lactate were measured using Zell Bio Lactate (ELISA) kits (CAT No. ZB-LAC-96A); in this method, lactate oxidase breaks lactate into pyruvate and hydrogen peroxide, which reacts in the presence of peroxidase with 4-aminoantipyrine and TBHB to produce a red chinonimin dye, an increase of color in which is proportional to lactate concentration. Serum NO levels were measured by the Griess method using a commercial kit (kiazist, Iran). Serum VCAM-1 levels were measured on the basis of biotin double antibody sandwich technology by commercial ELISA kits (Zell Bio, Germany; Cat. No: ZB-10203C-H9648).

### Statistical analysis

Statistical analyses were performed using SPSS for Windows version 20 (SPSS Inc., Chicago, IL, USA). P < 0.05 was considered statistically significant in all the analyses. The Q–Q plots, skewness statistics, and Shapiro–Wilk test were used to judge the normal distribution of the variables. The logarithmic transformation approach was applied for those markers with an abnormal distribution. For accurate evaluation of the differences between the two groups, a two-sided, two-sample, equal variance t-test was applied. Dietary intakes and physical activity were analyzed using a paired t-test. Numeric, normal variables were stated as means (SE). Within-group variances pre-and post- intervention were assessed by the paired t-test. To estimate the effects of ALA on the serum levels of the variables, covariance analysis (ANCOVA) was used, considering the impact of possible confounders (age, physical activity, total energy intake, marital status, educational status, economic status, and Gabapentin from drugs). All analyses were performed according to the Per-Protocol (PP) method, so only participants who completed the 3 months study period were entered into the analysis (compliance rate ≥ 90%).

## Results

### Baseline characteristics

**Figure 1:**
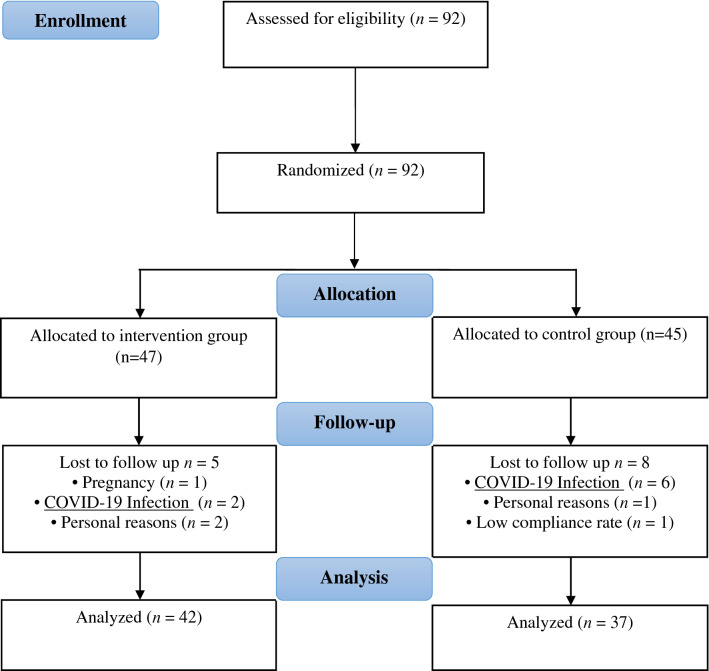
Flow diagram of the study based on CONSORT statement.

As displayed in Fig. 1, 92 women with episodic migraine were enrolled in the present randomized clinical trial, and 79 patients finished the study. 13 subjects (5 in the intervention and 8 in the placebo group) dropped out. In the intervention group, 5 patients discontinued the study due to infection with COVID-19 (n = 2), personal reasons (n = 2), and pregnancy (n = 1). In the placebo group, 8 patients were excluded due to COVID-19 infection (n = 6), personal reasons (n = 1), and low compliance (n = 1). All participants were between 20 and 50 years old. The baseline anthropometric and demographic features of the patients included in the final analysis are shown in Table 1. There was no significant difference between the ALA and placebo groups in terms of anthropometric measurements. Moreover, physical activity levels, drugs, and total energy intake were not significantly different. Marital (p = 0.023) and economic (p = 0.001) status were significantly different between the two groups.

**Table 1:** Baseline characteristics of participants (n = 92).

Variables	Intervention (n = 47)	Control (n = 45)	p
Age (years)	40.28 ± 1.291	43.31 ± 1.15	0.084
Weight (kg)	66.771 ± 1.70	69.155 ± 1.77	0.334
Height (cm)	161.363 ± 0.93	160.077 ± 0.85	0.314
Body mass index (kg/m^2^)	25.743 ± 0.70	27.022 ± 0.70	0.204
Physical activity (MET- min/week)	738.85 ± 81.71	676.288 ± 88.19	0.604
**Marital status, n (%)**			0.023
Married	36(76.6)	33 (73.3)	
Single	9 (19.1)	3 (6.7)	
Death of spouse or divorce	2(4.3)	9 (20)	
**Education status, n (%)**			0.050
Under diploma	14 (29.8)	21 (46.7)	
Diploma	14 (29.8)	16 (35.65)	
University	19 (40.4)	8 (17.8)	
**Economic status, n (%)**			0.001
Poor	4 (8.5)	7 (15.6)	
Moderate	20 (42.6)	32 (71.1)	
Good	23 (48.9)	5 (11.1)	
Very good	0 (0)	1 (2.2)	
**Job, n (%)**			0.890
Housewife	38 (78.7)	35 (77.8)	
Freelance	6 (12.8)	7 (15.6)	
Employee	4 (8.5)	3 (6.7)	
**Drugs, n (%)**			
Nonsteroidal anti-inflammatory drug (NSAIDs)	27 (57.4)	24 (53.3)	0.692
Beta blockers	6 (12.8)	6 (13.3)	0.936
Tricyclic Antidepressants (TCAs)	11 (23.4)	12 (26.7)	0.718
Tetracyclic antidepressants (TeCAs)	1 (2.1)	0 (0)	0.245
Topiramate	1 (2.1)	0 (0)	0.245
Sodium valproate	7 (14.9)	11 (24.4)	0.248
Gabapentin	6 (12.8)	1 (2.2)	0.057
Benzodiazepine	3 (6.4)	1 (2.2)	0.317
Triptans	4 (8.5)	1 (2.2)	0.169
Selective serotonin reuptake inhibitor (SSRIs)	1 (2.1)	0 (0)	0.245

The results of quantitative variables are presented as mean ± SE.

The results of qualitative variables are presented as, n (%).

P resulted from independent t-test.

### Dietary intakes

The dietary intake of the participants at baseline and post-intervention are shown in Table 2. Based on the 3-day food records, no significant intergroup differences were found in the dietary intakes for energy, carbohydrates, proteins, fat, micronutrients, and antioxidants.

**Table 2:** Dietary intakes of participants, obtained from two dietary records, throughout the study (n = 79).

	Intervention (n = 42)	Control (n = 37)	P
**Macronutrients**			
Energy (kcal/day)	1719.44 ± 37.91	1784.85 ± 27.47	0.164
Carbohydrate (g/day)	237.42 ± 8.74	241.78 ± 11.14	0.759
Protein (g/day)	63.52 ± 2.05	61.23 ± 1.7	0.395
Fat (g/day)	59.69 ± 2.35	67.72 ± 3.76	0.073
**Micronutrients**			
Cholesterol (mg/day)	208.08 ± 13.93	180.22 ± 7.92	0.084
Sodium(mg/day)	2203.18 ± 86.33	2128.46 ± 110.97	0.596
Potassium (mg/day)	2544.59 ± 107.05	2413.98 ± 74.45	0.318
Calcium (mg/day)	870.84 ± 48.21	764.97 ± 34.95	0.077
Magnesium (mg/day)	217.05 ± 10.9	223.97 ± 6.49	0.587
Folate (μg/day)	415.30 ± 16.29	464.17 ± 23.45	0.089
Vitamin C (mg/day)	98.26 ± 6.46	87.57 ± 4.34	0.172
Vitamin E (mg/day)	6.37 ± 0.45	6.11 ± 0.44	0.682

^1^All values are means ± SE.

P resulted by independent t-test.

### Clinical signs of migraine

The effects of ALA supplementation on migraine symptoms are to be seen in Table 3. The severity, frequency, duration, HDR, HIT-6, and MHIS were not significantly different between the two groups at the beginning of the study. Following supplementation with ALA for 3 months, significant reductions in severity (p < 0.001), frequency (p = 0.001), HDR (p = 0.003), HIT-6 (p < 0.001), and MHIS (p < 0.001) were found, but duration (p = 0.303) was not significantly different between the two groups. It should be noted that in the ALA group, all clinical signs, HDR, HIT-6 and MHIS showed a significant decrease compared with the beginning of the study (P < 0.001, for all). Variations in the placebo group were significant for severity, duration, and HIT6 (p = 0.023, p = 0.003, and p = 0.049 respectively), but changes in other variables were not significant in this group.

**Table 3:** The effects of Alpha-lipoic acid supplementation on migraine symptoms.

	Intervention (n = 42)	Control (n = 37)	P1	P3
**Severity**				< 0.001
Baseline	8.202 ± 0.25	8.022 ± 0.31	0.657	
End	4.72 ± 0.35	7.16 ± 0.40	< 0.001	
Mean change(CI)	− 3.59 (− 4.35, − 2.83)	− 0.70 (− 1.30, − 0.104)		
P2	< 0.001	0.023		
**Frequency**^**1**^				0.001
Baseline	5.723 ± 0.43	5.733 ± 0.57	0.989	
End	3.34 ± 0.39	5.05 ± 0.67	0.033	
Mean change	− 2.55 (− 3.24, − 1.87)	− 0.40 (− 1.61, 0.80)		
P2	< 0.001	0.502		
**Duration (h)**^**2**^				0.303
Baseline	36.957 ± 4.50	42.872 ± 4.40	0.351	
End	15.004 ± 3.62	26.06 ± 4.74	0.064	
Mean change	− 19.49 (− 29.49, − 9.49)	− 15.37 (− 25.19, − 5.54)		
P2	< 0.001	0.003		
**HDR**^**3**^				0.003
Baseline	205.851 ± 31.92	251.844 ± 43.98	0.397	
End	48.13 ± 11.55	195.66 ± 48.90	0.005	
Mean change	− 158.79 (− 223.38, − 94.20)	− 38.63 (− 122.39, 45.12)		
P2	< 0.001	0.356		
**HIT6**^**4**^				< 0.001
Baseline	70.383 ± 0.91	69.600 ± 1.37	0.633	
End	50.65 ± 1.76	67.27 ± 1.71	< 0.001	
Mean change	− 20.09 (− 23.81, − 16.36)	− 2.83 (− 5.65, − 0.015)		
P2	< 0.001	0.049		
**MHIS**^**5**^				< 0.001
Baseline	77.867 ± 13.03	83.32 ± 16.97	0.482	
End	15.006 ± 3.37	82.99 ± 15.74	< 0.001	
Mean change	− 65.32 (− 92.61, − 38.02)	− 0.33 (− 29.61, 28.93)		
P2	< 0.001	0.981		

P1 resulted from independent t-test, P2 resulted from paired sample t-test, P3 resulted from analysis of covariance in the adjusted models (adjusted for baseline level of each Variable, age, BMI, Marital status, Education status, Economic status, Gabapentin from drugs, Physical activity and energy intake); All values are means ± SE.

^1^Frequency of attacks per month.

^2^Average duration of migraine attack (hr).

^3^Headache dairy results: Duration of headache (hr) × frequency of headache.

^4^Headache Impact Test-6.

^5^Migraine Headache Index Score: Duration of headache (day) × frequency of headache × Severity.

### Biochemical measurements

**Figure 2:**
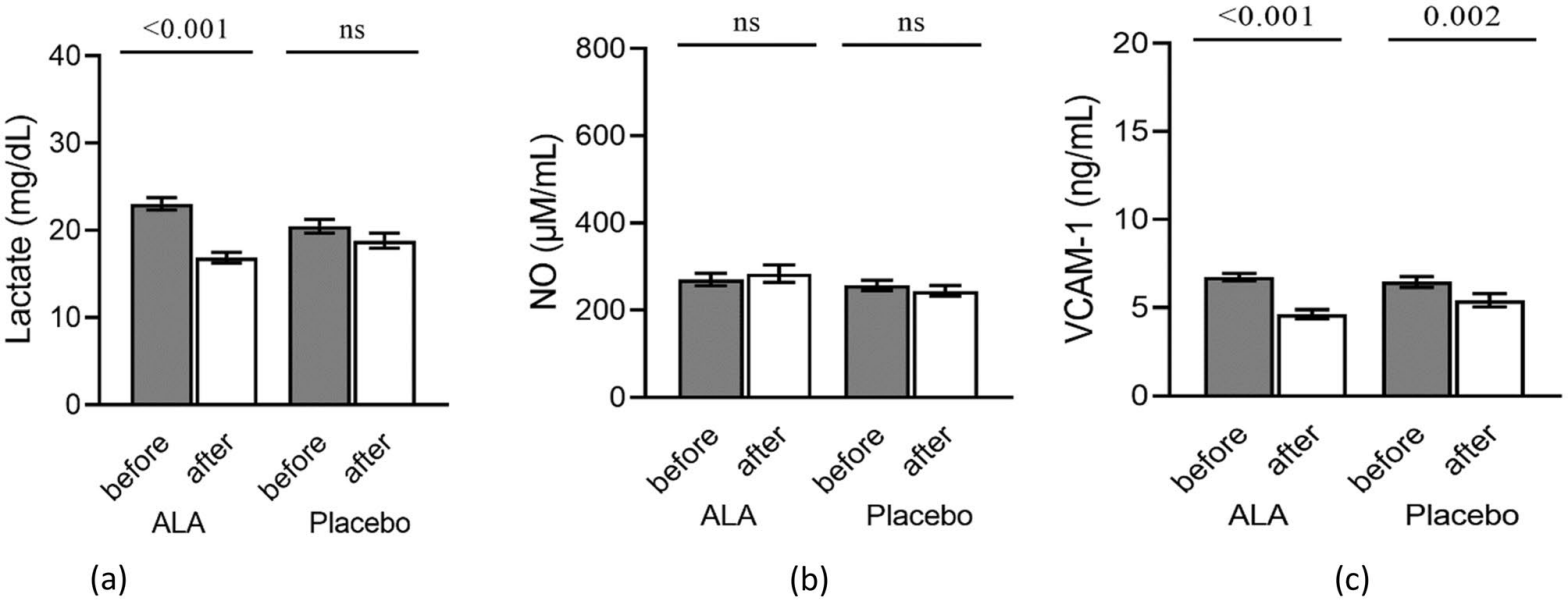
Comparison of biochemical variables between alpha-lipoic acid and placebo groups before and after the intervention. P resulted from paired sample t- test. (**a**) Lactate, (**b**) Nitric oxide (NO), (**c**) Vascular cell adhesion molecule-1 (VCAM-1).

The impacts of ALA supplementation on the biochemical variables in the female patients with episodic migraine are summarized in Table 4 and Fig. 2. No significant differences were observed in the baseline levels of NO and VCAM-1 between the two groups, while the baseline levels of lactate were significantly different (p = 0.018). After 3 months of intervention, and after adjustments for the baseline levels of the confounder variables including age, BMI, marital status, educational status, economic status, drugs, physical activity, and total energy intake, ALA supplementation significantly decreased serum levels of lactate and VCAM-1 in the ALA as compared with the placebo group (p = 0.039 and p = 0.025, respectively). Within-group analyses showed that lactate serum levels significantly decreased post-intervention only in the ALA group (p < 0.001), and VCAM-1 levels decreased in both groups (p < 0.001 in the ALA and p = 0.002 in the control group). No significant change was observed for NO levels.

**Table 4:** The effects of Alpha-lipoic acid supplementation on mitochondrial metabolic disorders marker, and vascular markers.

	Intervention (n = 42)	Control (n = 37)	P1	P3
**Lactate (mg/dl)**				0.039
Baseline	23.02 ± 0.71	20.47 ± 0.78	0.018	
End	16.87 ± 0.60	18.82 ± 0.85	0.062	
Mean change(CI)	− 6.45 (− 8.11, − 4.79)	− 2.27 (− 4.66, 0.107)		
P2	< 0.001	0.061		
**NO (µM/ml)**				0.104
Baseline	270.15 ± 14.42	256.78 ± 11.56	0.474	
End	283.69 ± 20.22	243.89 ± 12.54	0.099	
mean change	15.75 (− 34.03, 65.54)	− 15.37 (− 44.49, 13.75)		
P2	0.526	0.292		
**VCAM1(ng/ml)**				0.025
Baseline	6.72 ± 0.22	6.45 ± 0.30	0.480	
End	4.63 ± 0.26	5.41 ± 0.37	0.088	
Mean change	− 2.02 (− 2.064, − 1.40)	− 1.21 (− 1.95, − 0. 47)		
P2	< 0.001	0.002		

P1 resulted from independent t-test, P2 resulted from paired sample t-test, P3 resulted from analysis of covariance in the adjusted models (adjusted for baseline level of each Variable, age, BMI, Marital status, Education status, Economic status, Drugs, Physical activity and energy intake); All values are means ± SE.

Some side effects reported by patients include stomach pain (6.3% in the intervention group versus 8.8% in the placebo group), increased appetite (4.2% in the intervention group versus 2.2% in the placebo group), and constipation (4.4% in the placebo group).

## Discussion

The results of the present study showed that 3 months of ALA supplementation in women with episodic migraines led to a significant reduction in serum lactate and VCAM-1 levels. Moreover, HDR, HIT6, MHIS, and migraine symptoms, including severity and frequency, were significantly reduced in the ALA as compared to the control group. However, changes in serum NO levels and duration of migraine pain were not statistically significant.

Although the mechanism of migraine pathogenesis is not completely understood, it is supposed that hypoxia or mitochondrial dysfunction is involved in its pathogenesis^11^. Mitochondria play a fundamental role in the functions of neurons through producing adequate ATP and regulating intracellular calcium levels^14^. MRS studies in migraine consistently show abnormalities of mitochondrial function such as hypo-metabolism or decreased ATP levels^11,69^. Numerous studies have proved that lactic acid levels are increased in migraine patients^48^. So, elevated levels of lactate are proposed as an indicator of defective oxidative metabolism^12,14^. Hypoxia is followed by an increase in CBF, NO, and FMD in migraine^22,27,32^. It should be noted that NO production is one of the mechanisms involved in the cortical spreading depression (CSD)-induced alterations of the cerebrovascular responses^35^. The activation of CSD results in the increase of ICAM-1 and VCAM-1expression^35^. It has been reported that the rises of ICAM-1 and VCAM-1 in the endothelial cells are positively associated with an escalation in transendothelial transportation^70^. The release of pro-inflammatory mediators via the engaged immune cells further magnifies meningeal afferent sensitization^71^. It is suggested that vasodilation and inflammation induced by endothelial dysfunction contribute to meningeal afferent sensitivity and migraine pain^71^. It is expected that improving mitochondrial function and hypoxia status would effectively reduce migraine symptoms by modulating endothelial function and reducing inflammation. The available evidence suggests that consumption of some nutrients such as riboflavin^44,46^, coenzyme Q10^47,48^, magnesium^45^ and ALA^60–62^ have been shown to improve the clinical symptoms of migraine by improving mitochondrial function.

In this study, we observed that ALA supplementation leads to a reduction in serum lactate levels. Elevated lactate levels in migraine sufferers have been shown in previous studies^48,72,73^; But a study by Gross et al. reported different results. According to the study, only two migraine patients had high baseline lactate levels^59^, a result which was in contrast with other studies^18,20,21^. None of the previous studies have investigated the effect of ALA supplementation on serum lactate levels. ALA is an important cofactor for mitochondrial metabolism and is an essential cofactor for catalysis by some mitochondrial enzymes, including pyruvate dehydrogenase, α-ketoglutarate dehydrogenase^74^. Also, ALA displays a critical role in stabilizing and regulating these multi-enzyme complexes. Therefore, by increasing mitochondrial function, ALA can reduce lactate levels and increase ATP production^74^.

This study indicated that supplementation with 600 mg/day ALA for 3 months, did not bring about significant changes in the mean levels of serum NO. NO is synthesized from L-arginine by three isozymes of nitric oxide synthase (NOS), containing neuronal NOS (nNOS), endothelial NOS (eNOS), and inducible NOS (iNOS)^75^. The role of NO in the pathogenesis of migraine has been proven in previous studies^24^. None of the previous trials have measured serum NO levels in the evaluation of the influence of ALA on migraine^60–62^. The results for ALA supplementation are inconsistent. Badran et al. revealed that supplementation with ALA attenuates endothelial dysfunction by averting oxidative stress and inflammation and restoring NO bioavailability in mice exposed to chronic intermittent hypoxia (CIH)^76^.

On the other hand, another study demonstrated that ALA in pharmacologically related doses decreases the expression of some important inflammatory mediators, such as iNOS in rat Kupffer cells^77^. An increase in the intake of nitrate-containing foods before the final blood draw may be a possible explanation for the lack of significant changes in serum NO levels. As previously documented, serum NO levels are directly affected by nitrate-containing foods, as NO precursors^78^. As one of the weaknesses of this study, we did not examine the levels of intake of nitrate-containing foods.

In the case of the present study, the treatment of women suffering episodic migraines with ALA significantly decreased serum VCAM-1 concentration. None of the previous studies designed to investigate the effect of ALA intake on migraine had determined VCAM-1 levels. Neuroinflammatory processes and vasomotor changes are mediated by various neuropeptides and cytokines in migraine. A special pattern of inflammatory indicators has been detected in the systemic circulation in migraine patients, including increased levels of C-reactive proteins (CRP)^79^, interleukins (ILs e.g. IL-1 and IL-6)^80,81^, tumor necrosis factor-alpha (TNF-α) and adhesion molecules (ICAM and VCAM)^23,82^. These inflammatory markers disrupt the tendency of the blood cells to aggregate and lead to thrombosis and endothelial dysfunction^83,84^. Nilsson Remahl et al. showed that compared with a healthy control group, the mean levels of soluble adhesion molecules in Cluster Headache patients also tended to be higher, but statistically significantly so only for sVCAM-1^85^. In line with our result, in 1999, Kant et al. demonstrated that ALA decreases expression of VCAM-1 and endothelial adhesion of human monocytes after stimulation with advanced glycation end products^86^. The next study in 2006 showed that ALA impedes the expression of ICAM-1 and VCAM-1 by the central nervous system (CNS), endothelial cells, and T cell migration into the spinal cord in experimental autoimmune encephalomyelitis^87^. According to the results of previous studies, Ismawati et al. in 2019 showed that treatment with ALA in diabetic rats reduces both the oxidated low-density lipoprotein (oxLDL) levels in plasma and VCAM-1 expression on the aortas^55^. Thus, the results of our study are in line with previous studies. Since during the intervention the use of prophylaxis drugs continued in both the intervention and control groups, decrease in VCAM-1 levels may have been the result of reduced inflammation in the control group. However, the VCAM-1 intergroup changes are still significant, which confirms the protective effect of ALA on endothelial function. ALA exerts its antioxidant effects through the capture of ROS, the regeneration of endogenous antioxidants and the restoration of oxidized proteins, and modulates the transcription of genes and inhibits NF-*κ*B activation^55,88^.

We were able to show that prophylactic ALA treatment favorably affects migraine symptoms, including frequency (days/month) and severity of attack as well as HDR, HIT6, and MHIS, but the changes in the intergroup attack duration were not significant. Visual Analogue Scale (VAS) is a well-validated instrument, which is used to assess pain intensity^89^. The results are also in agreement with the findings in previous studies that ALA supplementation can decrease migraine severity and frequency. A study in 2007 showed that treatment with 600 mg/day ALA for 3 months significantly reduces migraine attack frequency, the number of headache days, and headache severity^60^. Ali et al. in 2010 showed that combined topiramate (50 mg/day)/ALA (300 mg/day) therapy meaningfully decreased mean monthly migraine frequency and attack duration compared to those receiving either topiramate or ALA only^61^. In another study in 2017, Cavestro et al. reported that the administration of ALA (400 mg b.i.d. for 6 months) might be associated with a reduction in the number of attacks and the days of treatment in migraineurs with insulin resistance^62^. Significant changes in attack duration in both the intervention and placebo groups following migraine medication could be a reason for the insignificance of the intergroup changes. HIT-6 is a short form of the Headache Impact Test questionnaire, which is widely used to assess the adverse effects of headaches on normal daily activities. The HIT-6 questionnaire consists of 6 items: that evaluate how often headaches are caused by severe pain, how often the headache limits daily activities, how often they makes you want to lie down, or how often they causes fatigue, irritability, and affects concentration. Each item can have one of 5 responses (Never, Rarely, Sometimes, Very often, or Always). After assigning the specified numerical value to each answer (6, 8, 10, 11, and 13, respectively), the sum of the scores can be a range of 36–78^90^. Analysis of our study data showed that supplementation with ALA reduced HIT-6 by 20 scores in intervention group, compared with about 3 scores in the control group. The results of the analysis show that despite the favorable effects of medication on migraine severity and the HIT-6 score in both groups, intergroup changes remained significant, indicating a strong prophylactic potential for ALA in migraine. As previously mentioned, the positive role of ALA in migraine prevention and treatment may be due to diverse mechanisms such as its function in mitochondrial energy production, antioxidant and anti-inflammatory effects^55,74^.

The present study has several advantages. To the best of our knowledge, this study is the first research that was designed to assess the effects of ALA on endothelial markers such as NO and VCAM-1. Also, proper blinding and controlling confounder elements such as baseline values, drugs received, BMI, and total energy intake can be noted. However, the study has some limitations. First, we did not measure the serum ALA concentration at baseline and at the end of the study due to limited financial resources. Therefore, we were not able to determine the extent of ALA deficiency in migraine patients. In addition, the patients' adherence was determined based on self-report and the counting of remaining capsules. Second, we did not assess intake of nitrate-containing foods, which could help interpret serum NO levels. Therefore, further studies with a longer duration may be required to confirm the health profits of ALA supplements in patients with migraine.

In conclusion, the findings of this study propose that ALA has beneficial effects on mitochondrial and endothelial function as well as clinical signs of migraine. Therefore, ALA may be considered as a potential adjunctive therapy in migraine.

## Data Availability

The datasets generated during and/or analyzed during the current study are available from the corresponding author on reasonable request.

## Abbreviations

ALA: Alpha-lipoic acid
NO: Nitric oxide
VCAM-1: Vascular cell adhesion molecule-1
HIT-6: Headache impact test-6
HDR: Headache dairy results
MHIS: Migraine headache index score
MA: Migraine with aura
MwoA: Migraine without aura
31P-MRS: Phosphorus magnetic resonance spectroscopy
CBF: Cerebral blood flow
FMD: Flow-mediated dilation
CSD: Cortical spreading depression
CAMs: Cellular adhesion molecules
ICAM: Intracellular adhesion molecules
HIS: International Headache Society
CNS: Central nervous system
